# Identification of Longitudinal Associations Between Depressive Symptoms and EEG Functional Connectivity in Adolescents Receiving Antidepressant Treatment

**DOI:** 10.21203/rs.3.rs-9044389/v1

**Published:** 2026-06-09

**Authors:** Wadim Vodovozov, Asif Jamil, Benjamin Wade, Joan Camprodon, Farren Briggs, Molly McVoy

**Affiliations:** Zucker Hillside Hospital; Massachusetts General Hospital, Harvard Medical School; Case Western Reserve University School of Medicine

## Abstract

Major depressive disorder (MDD) is the most common mood disorder in adolescents, affecting up to 20% of adolescents in the US. Identifying neurophysiological functional connectivity biomarkers that track treatment response and provide mechanistic insights could inform early interventions and improve depression outcomes in adolescents. Resting-state quantitative electroencephalography (qEEG) provides a low-cost, non-invasive method to examine functional connectivity and link connectivity dynamics to symptom changes during treatment. We utilized qEEG to identify associations between functional connectivity measures and depressive symptom severity over time by analyzing longitudinal data from 21 adolescents with moderate-to-severe MDD (85.7% female) recruited at University Hospitals Cleveland Medical Center, Ohio. Participants completed EEG recordings and depressive symptom assessments using the Children’s Depression Rating Scale-Revised (CDRS-R) at baseline (pre-treatment), week 4, and week 16. Participants received fluoxetine or escitalopram alongside evidence-based therapy (EBT). We computed baseline functional connectivity measures for selected channel pairs across five canonical EEG frequency bands. Principal component analysis (PCA) reduced the dimensionality of baseline functional connectivity metrics to seven components, which were projected onto subsequent time points. Longitudinal associations between PCs and CDRS-R Total and subscale scores were inspected with a linear mixed effects model (LMEM). We observed robust clinical improvement over time. PC2 and PC3 showed significant time-dependent interactions with CDRS total, while PC3 revealed a significant interaction with CDRS Morbidity over time. These findings suggest that fronto-parietal and fronto-temporal connectivity features, especially involving Weighted Pairwise Phase Consistency (WPPC) and Coherence, longitudinally track treatment response in adolescents undergoing antidepressant treatment.

## INTRODUCTION

Psychiatric disorders are among the leading causes of morbidity and mortality in adolescents in the United States. More than 14 million adolescents suffer from psychiatric illnesses that impair functioning and cause long-term suffering ^1^. Major depressive disorder (MDD) affects up to 20% of adolescents in the United States and leads to significant functional impairment in school and home environments ^2,3^. The impact of adolescent MDD can be severe, contributing to substance use disorders, self-injury, and suicide. In fact, suicide remains the second leading cause of death in U.S. adolescents, surpassing cancer, cardiovascular disease, birth defects, lung disease, and influenza combined ^4^. Currently, diagnostic approaches for MDD in adolescents rely primarily on rating scales, which, while generally reliable, remain subjective, may delay timely treatment initiation, and have limited sensitivity to treatment-related symptom changes. ^5,6^. Resting-state quantitative electroencephalography (qEEG) has emerged as a promising tool to fill this gap. It provides a low-cost, non-invasive method with high temporal resolution to examine neural functional connectivity patterns associated with symptom changes during treatment. Accumulating evidence links dysfunction of resting state networks to the development of mood and anxiety disorders in young adults ^7,8^. While many qEEG biomarkers have been investigated for MDD in adults ^9–12^, the data on pediatric MDD has been sparse to date. The substantial neurophysiological differences between developing adolescent and mature adult brains underscore the importance of adopting age-specific approaches for the identification of neurophysiological biomarkers of depression. In adolescents, depression has been more consistently associated with increased gray matter volume in the dorsolateral prefrontal cortex ^13^, with increased functional activation shown in neuroimaging studies ^14^. In contrast, adult depression appears to be more commonly linked to reduced gray matter volume in the orbitofrontal cortex, cingulate, insula, and temporal regions ^15,16^. The increased neural plasticity of adolescence provides a window of opportunity for early intervention. The study of neural functional connectivity dynamics using qEEG can yield mechanistic insights into antidepressant treatment response and serve as a diagnostic biomarker for adolescent depression, tracking treatment-related symptom changes. Previous work of our group with qEEG showed a correlation between both the diagnosis of depression and the severity of depressive symptoms with aberrant patterns of EEG coherence ^17,18^. We previously presented a unified predictive model for adolescent MDD which included coherence, power, and cross-correlation to differentiate adolescent MDD from healthy controls ^19^. We now expand our EEG analysis to include additional time points in order to assess longitudinal changes of functional connectivity measures. In this exploratory study, we aim to identify longitudinal correlations between EEG functional connectivity (FC) measures and depressive symptoms over the course of treatment, measured with *Children’s Depression Rating Scale-Revised (CDRS-R)* scores as well as additional subscores. We use an expanded set of EEG FC measures, namely *Coherence, Imaginary Coherence*, *Weighted Phase Lag Index (WPLI)*, and *Weighted Pairwise Phase Consistency (WPPC)* ^20–23^. The overarching goal of this study is to determine whether qEEG measures change with depressive symptoms over the course of treatment. We hypothesize that changes in specific FC measures are longitudinally associated with treatment-related changes in symptom severity in adolescent MDD.

## METHODS

### Study Design

This exploratory prospective cohort study analyzed longitudinally collected EEG and psychometric data of a cohort of 21 adolescents diagnosed with moderate-to-severe MDD. Depressive symptoms were assessed at baseline, week 4, and week 16 using the Children’s Depression Rating Scale-Revised (CDRS-R) including some of its inherent subscores ^24^. EEG data were collected during the same three visits. All participants received fluoxetine or escitalopram in combination with evidence-based therapy (EBT).

### Participants

The study and all recruitment materials, including physical and social media advertisements, were approved by the local Institutional Review Board of University Hospitals Cleveland Medical Center. Written informed consent was obtained from all caregivers and assent of all participants prior to enrollment. Recruitment for the study involved referrals from clinical providers within the department of psychiatry at an urban outpatient clinic, and community outreach through printed study flyers. Participants were required to meet the diagnostic criteria for MDD as outlined in the *Diagnostic and Statistical Manual of Mental Disorders, 5th edition* (DSM-5; American Psychiatric Association, 2013), based on an initial intake evaluation. The diagnosis was further confirmed using the Mini International Neuropsychiatric Interview for Children and Adolescents (MINI-KID) ^25^. Participants needed to have a score of 40 or higher on the CDRS-R, indicating at least moderate MDD severity. Exclusion criteria for the MDD group included a current or past diagnosis of bipolar or any psychotic disorder, a history of brain surgery involving implanted devices or shunts, a history of seizure disorder with the exception of febrile seizures, or an active or current diagnosis of meningitis.

### EEG Data Acquisition & Processing

EEG data were collected using a Nihon Kohden EEG-1200 system, which has been approved by the U.S. Food and Drug Administration for clinical applications. Resting-state EEG recordings were conducted in a sound-attenuated room while participants lay quietly with their eyes closed. To minimize drowsiness, participants were periodically alerted and instructed to remain still, avoiding excessive blinking or eye movements throughout the recording session. EEG signals were captured using the Discovery 24 E amplifier, a 32-channel 10–20 EEG system. The system utilized an initial average reference based on the C3 and C4 electrodes, with a common average re-referencing conducted offline. The recorded EEG channels included Fp1, Fp2, F7, F8, T7, T8, P7, P8, O1, O2, F3, F4, C3, C4, P3, P4, Fz, Cz, and Pz. Additional channels, including LSO, RSO, LIO, RIO, LOC, and ROC, were also acquired originally to accurately monitor and account for eye movements. To facilitate dimensionality reduction, the pipeline was optimized with a reduced set of nine non-adjacent channels, while also accounting for whole brain coverage. The remaining 9 channels were Cz, Fz, Pz, F7, F8, T7, T8, P7, and P8, which led to 72 possible channel dyad combinations, including redundant dyads.

### EEG Data Analysis

EEG data were bandpass filtered (0.3–50 Hz) to remove slow drifts and high-frequency noise. Artifacts were identified using Artifact Subspace Reconstruction (ASR), an automated algorithm implemented in EEGLAB in MATLAB, to identify and remove high-variance artifacts and noisy channels, which were then repaired using spline interpolation ^26^. To further mitigate artifacts, independent component analysis (ICA) was performed to remove contributions from eye movements, cardiac activity, and muscle artifacts. After artifact removal, the data were re-referenced to the average of all channels and then segmented into 4-second epochs with 50% overlap, ensuring sufficient temporal resolution for spectral and connectivity analyses. To further eliminate residual extreme artifacts, segments exceeding a 150 μV amplitude threshold were automatically rejected. Following preprocessing, four functional connectivity features were extracted using phase- and coherence-based measures. While traditional coherence metrics are highly susceptible to artifacts from volume conduction and reference electrode effects, leading to spurious detection of connectivity, phase-based measures and imaginary coherence have been developed to effectively reduce the influence of zero-lag correlations, which are most likely to arise from volume conduction rather than genuine neural communication. Coherence itself still remains a valid measure for assessing functional connectivity due to its mathematical relationship with cross-spectral density of the power spectrum and its ability to capture phase consistency between signals. Thus, the following 4 functional connectivity measures were calculated for each electrode dyad across five frequency bands, delta (1–4 Hz), theta (5–7 Hz), alpha (8–12 Hz), beta (13–30 Hz), and gamma (31–45 Hz). 1. *Coherence (Coh)* was calculated to estimate the linear correlation between signals across electrode pairs; *2. Imaginary Coherence (Imag Coh)* was extracted to reduce volume conduction effects and capture genuine long-range interactions; 3. *Weighted Phase Lag Index (WPLI)* was computed to quantify phase synchronization while minimizing spurious connectivity; 4. *Weighted Pairwise Phase Consistency (WPPC)* was calculated to assess the stability of phase relationships over time ^20–23^.

### Symptom Assessment

Depressive symptom severity was assessed using the clinician-rated Children’s Depression Rating Scale-Revised (CDRS-R) at baseline (pre-treatment), week 4, and week 16. The chosen subscores, derived from CDRS-R, comprised CDRS *Morbidity*, CDRS *Suicidality*, CDRS *Difficulty having fun*, and CDRS *Social withdrawal*. The morbidity subscore reflects overall illness severity and the impact of depressive symptoms on daily functioning; the suicidality subscore captures the presence and intensity of suicidal thoughts or behaviors; the difficulty-having-fun subscore assesses anhedonia in age-appropriate activities; and the social withdrawal subscore measures reduction in social engagement and interpersonal interactions.

### Statistical Analysis

Principal component analysis (PCA) was performed in R Studio (version 2025.05.0 + 496) with R (version 4.4.2) on 1440 pre-processed EEG functional connectivity features derived from all 72 redundant channel dyads across five frequency bands and four connectivity metrics, Coh, Imag Coh, WPLI, and WPPC, at Session 1 (*Baseline*) from the original 28 participants with MDD. PCA involves computing the covariance matrix to capture relationships between variables, followed by eigen decomposition to extract eigenvectors (PCs) and corresponding eigenvalues, which indicate the proportion of variance explained by each PC. EEG functional connectivity data at Session 2 (*Week 4*) and Session 3 (*Week 16*) from the 21 participants with complete EEG and CDRS data across all three sessions were projected onto the baseline-derived PCA loadings. This led to 21 PCs capturing maximal variance in the EEG connectivity data with fewer dimensions. This approach ensured projection of all EEG connectivity data into a common PCA space while preserving the baseline-derived component structure. This enabled a consistent interpretation of EEG connectivity changes over time. Each component accounted for a progressively smaller portion of the variance. We retained the first 7 components as they collectively explained 53.24% of the total variance. While this proportion falls below conventional thresholds used in some fields, this dimensionality was selected based on a balance between capturing shared signal structure and reducing the risk of overfitting in subsequent mixed-effects models. Given the high collinearity and noise inherent in EEG data, retaining the top seven components provided a robust summary of dominant connectivity patterns across subjects. A seven-component PCA solution in qEEG data has been shown to yield high internal consistency and test–retest reliability, indicating that these components are both stable and meaningful across subjects ^27^. Linear mixed-effects models (LMEMs) were constructed using the lmer() function from the lme4 package in R. First, we fit a model with *Session* (*Baseline*, *Week 4*, *Week 16*) as a categorical factor and *CDRS Total* score as the dependent variable, including a subject-level random intercept. This allowed fixed-effect contrasts to compare symptom severity between all pairwise time points without assuming a linear trajectory of change. In a separate LMEM, *Session* was treated as a continuous variable to examine longitudinal associations between EEG connectivity patterns and depressive symptoms. Seven baseline-derived *PCs* and their interactions with *Session* were entered as fixed effects, with *Age* as a covariate, *CDRS Total* or *Subscale* scores as the outcome, and a subject-level random intercept. *PC* × *Session* interactions were interpreted as indicators of whether specific EEG connectivity patterns tracked symptom change over time. Given the exploratory nature of the analysis, p-values were not adjusted for multiple comparisons to prioritize sensitivity in detecting symptoms of interest. Given the small number of male participants, we did not include *Sex* as a covariate as it would potentially risk model instability and overfitting. In addition, the data for participants using either fluoxetine or escitalopram was not available for all patients and was therefore not used as a covariate either.

### Results

**Figure 1:**
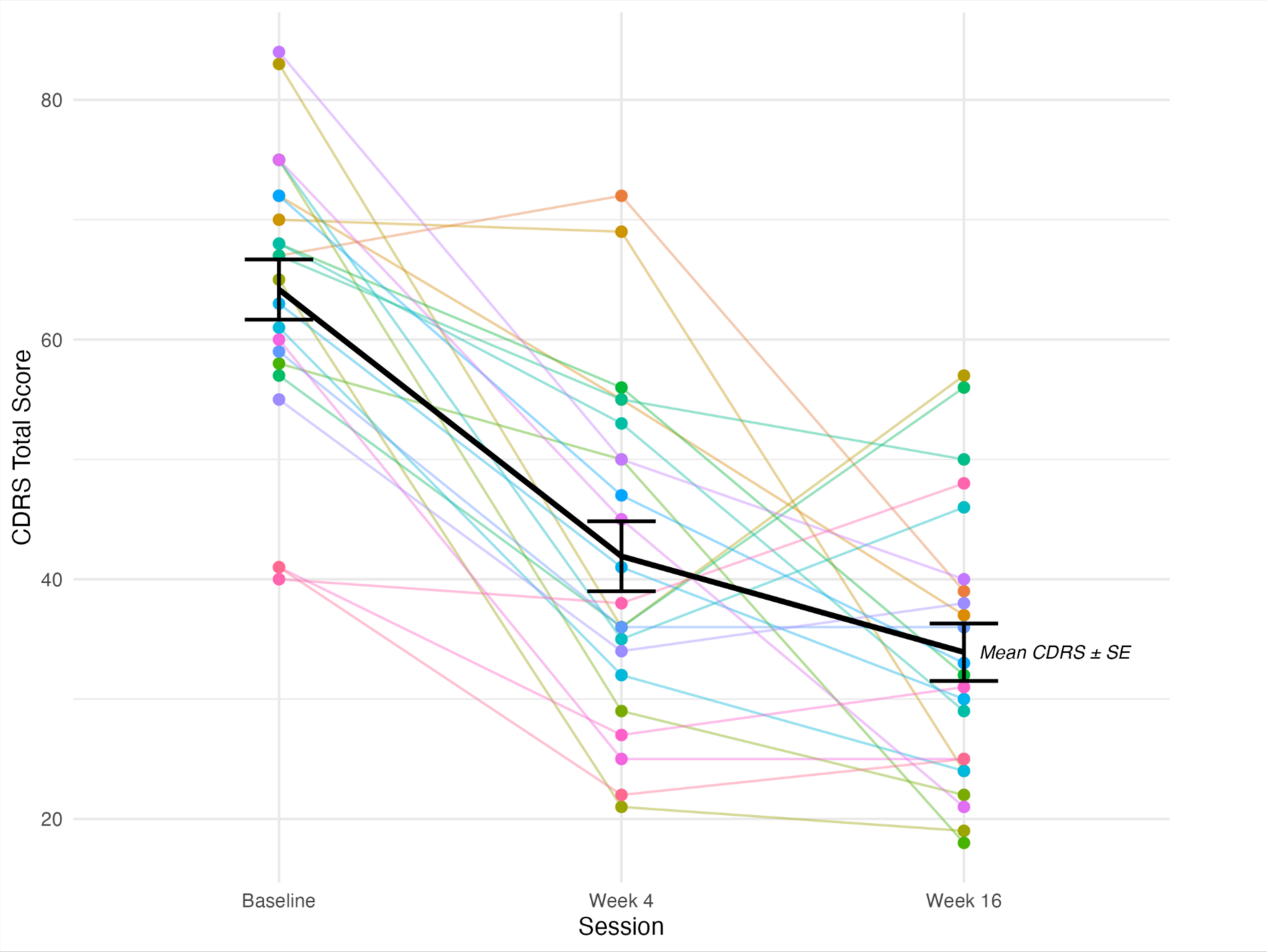
CDRS Total Scores over time. Each colored line represents an individual participant’s CDRS-R total score across three time points: *Baseline, Week 4, and Week 16*. The thick black line shows the group mean trajectory, and vertical bars represent the standard error (SE) at each session. A sharp reduction in depressive symptoms is evident between *Baseline* and *Week 4*, followed by a plateau phase from *Week 4* to *Week 16*.

Demographics along with clinical scores are presented in Table 1. CDRS scores of 21 adolescents with moderate-to-severe MDD (mean age = 15.71 [SD = 1.15], 86% female) over the three sessions (*Baseline*, *Week 4* and *Week 16*) revealed decreasing CDRS scores over time (see Fig. 1). A linear mixed-effects model (LMEM) using *Session* as a factor and *CDRS Total* score as the dependent variable revealed a significant main effect of time on CDRS scores. Tests of fixed effects contrasts from the LMEM showed that, compared to *Baseline*, CDRS scores significantly decreased from *Baseline* to *Week 4* (β = −23.14, SE = 3.35, t(40) = −6.91, p < 0.001) and from *Baseline* to *Week 16* (β = −29.10, SE = 3.35, t(40) = −8.69, p < 0.001). The estimated random intercept variance across subjects was 38.79 (SD = 6.23), and residual variance was 117.69 (SD = 10.85), indicating meaningful inter-individual symptom score variability. The reduction from Baseline to Week 4 yielded a Cohen’s d = −2.13 (95% CI: [−2.76, −1.51]), while the reduction from Baseline to Week 16 yielded a Cohen’s d = −2.68 (95% CI: [−3.31, −2.06]). The model showed a marginal R^2^ of 0.506 and a conditional R^2^ of 0.628. On the other hand, a direct comparison between Week 16 and Week 4 only showed a trend reduction in CDRS scores (β = −5.95, SE = 3.35, t(40) = −1.78, p = 0.083, Cohen’s d = −0.55, 95% CI: [−1.17, 0.08]).

### Longitudinal Associations Between CDRS Total Score and EEG Connectivity Features

**Figure 2:**
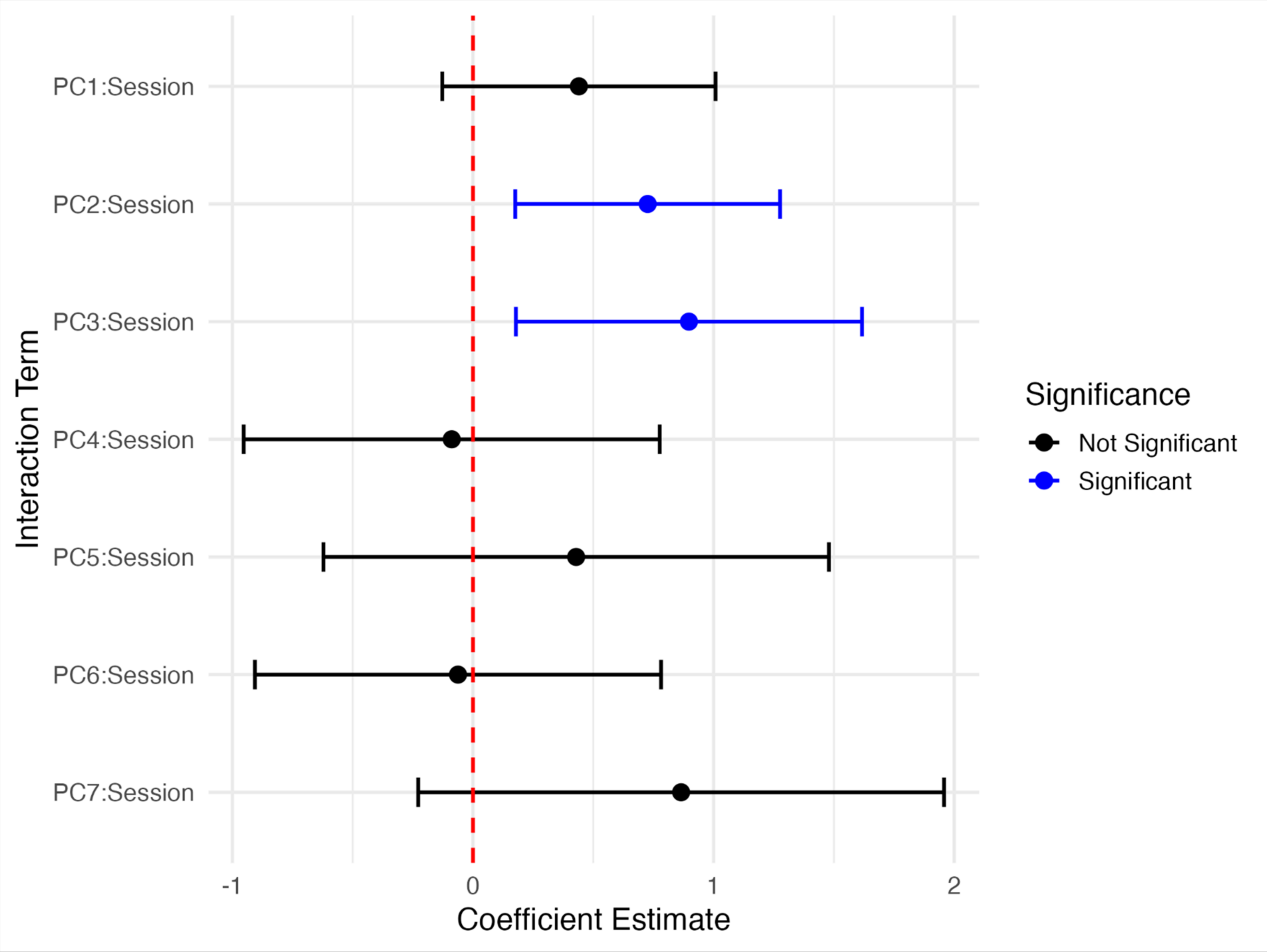
Forest Plot of PC x Session interaction terms on CDRS Total. Each point represents the estimated interaction effect between a principal component (*PC1–PC7*) and *Session* (*Baseline, Week 4, Week 16*) on *CDRS Total*. Horizontal lines show 95% confidence intervals, the red dashed line indicates the null effect. See significant interaction terms for PC2 and PC3 in blue: greater expression of PC2 and PC3 was associated with smaller reductions of CDRS scores over time.

To examine how EEG connectivity features captured by PCs were associated with changes in depressive symptoms over time, we fit a LMEM with *CDRS Total* score as the dependent variable. The fixed effects included *PC1–PC7, Session* (*Baseline, Week 4, Week 16*), and *PC × Session* interaction terms. We observed trend level main effects for PC2 (*β* = −0.905, SE = 0.536, *t* = −1.687, *p* = 0.098) and for PC3 (*β* = −1.002, SE = 0.580, *t* = −1.729, *p* = 0.090). This suggests that higher overall values on PC2 and PC3 may be modestly associated with lower average CDRS scores across sessions potentially reflecting trait-like variability rather than longitudinal symptom change. As shown in Fig. 2, PC2 and PC3 showed statistically significant interactions with *Session*, indicating time-dependent relationships with depression severity. PC2 × Session (β = 0.726, SE = 0.274, t = 2.65, p = 0.011) and PC3 × Session (β = 0.898, SE = 0.358, t = 2.51, p = 0.017) showed significant positive interactions, indicating that higher PC2 and PC3 values were associated with less reduction in CDRS scores over 16 weeks. This may imply that participants with higher PC2 or PC3 values showed a weaker treatment response. All other *PC × Session* interactions were nonsignificant.

### Longitudinal Associations Between CDRS Subscales and EEG Signals

**Figure 3:**
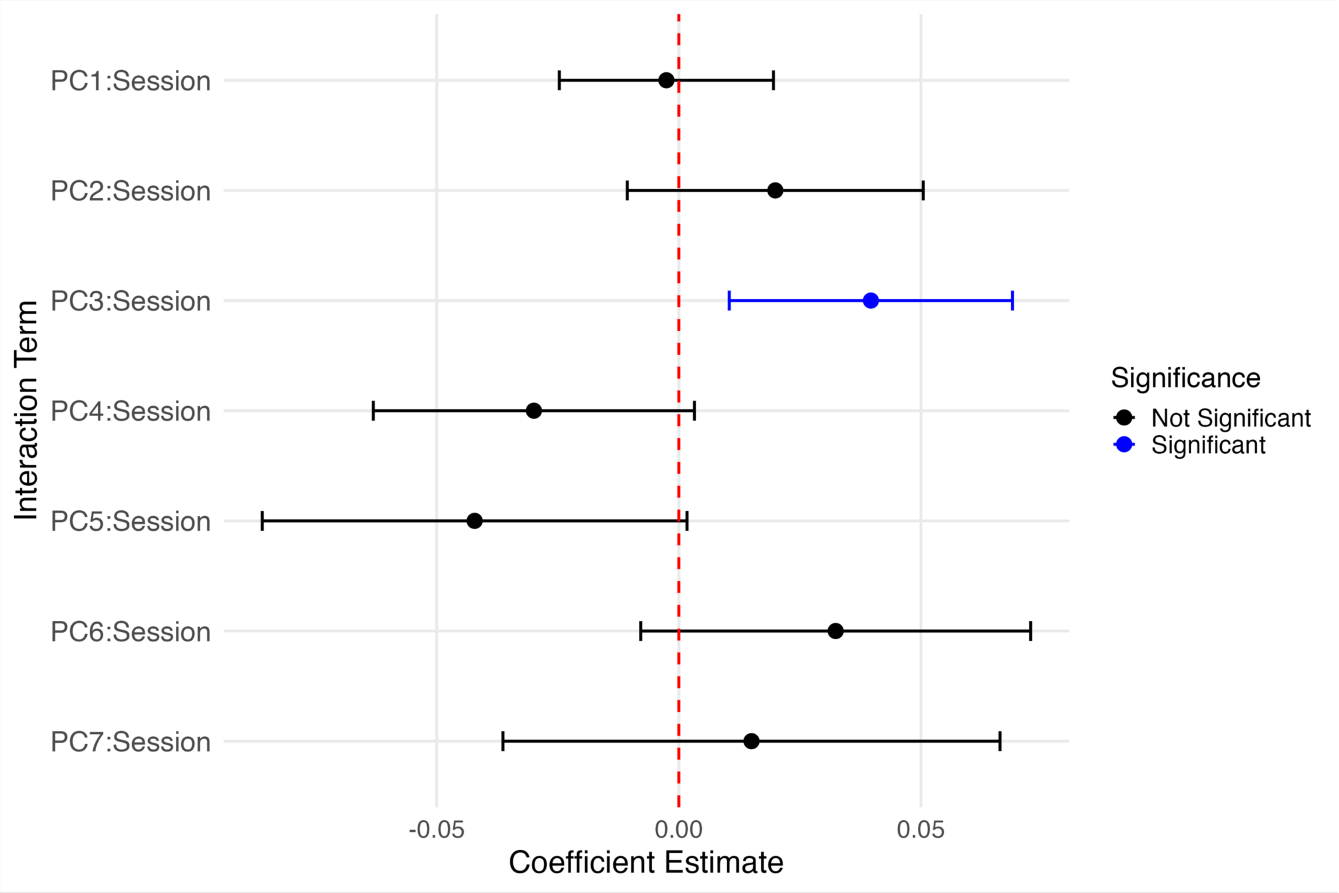
Forest Plot of PC x Session interaction terms on CDRS Morbidity. As in Fig. 2, each point represents the estimated interaction effect between a principal component (*PC1–PC7*) and *Session*, but the dependent variable is the *CDRS Morbidity* subscore. The significant interaction term for PC3 is highlighted in blue: greater expression of PC3 was associated with smaller reductions in *CDRS Morbidity* subscores over time.

Trend level associations were observed for the main effect of PC1 on CDRS *Difficulty having fun* (β = 0.048, SE = 0.029, *t* = 1.685, *p* = 0.099) and CDRS *Social withdrawal (*β = 0.055, SE = 0.029, *t* = 1.886, *p* = 0.066*).* As shown in Fig. 3, The interaction of PC3 x Session was significantly associated with the CDRS *Morbidity* subscale *(*β = 0.040, SE = 0.014, *t* = 2.756, *p* = 0.009*)*. Significant main effects for CDRS *Morbidity were* observed for PC3 (β = −0.099, SE = 0.034, *t* = −2.940, *p* = 0.005), PC4 (β = 0.081, SE = 0.038, *t* = 2.141, *p* = 0.038), PC5 (β = 0.146, SE = 0.054, *t* = 2.721, *p* = 0.009), and PC6 (β = −0.110, SE = 0.046, *t* = −2.383, *p* = 0.021). Trend level longitudinal interactions effects were observed for CDRS *Morbidity* for PC4 (β = −0.030, SE = 0.016, *t* = −1.835, *p* = 0.075) and PC5 (β = −0.042, SE = 0.022, *t* = −1.954, *p* = 0.059). In contrast, CDRS *Suicidality* showed no significant main effects or interactions.

### Spatial Distribution of Functional Networks Captured by Significant PCs

**Figure 4:**
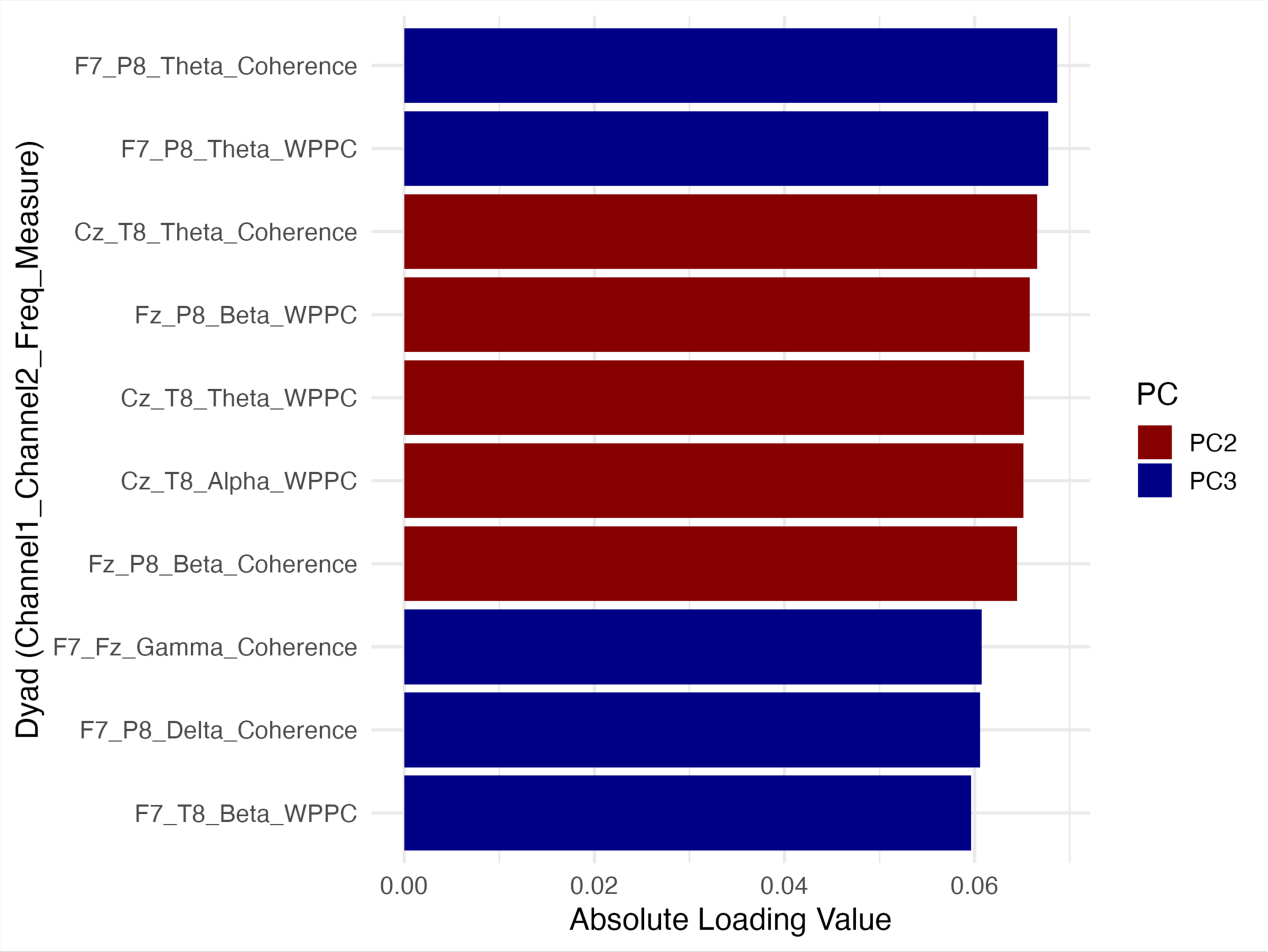
Top 5 Dyads for PC2 and PC3. Bar plots show the top five channel-frequency connectivity features with the highest absolute loading coefficients for *PC2* (red), and *PC3* (blue) that were significantly associated with changes of *CDRS Total* and *CDRS Morbidity*. PC2 captured multi-frequency WPPC and coherence features in fronto-parietal networks, while PC3 reflected similar WPPC and coherence patterns extending to fronto-temporal networks.

To further characterize the neurophysiological underpinnings of the PCs linked with changes in depressive symptoms, we examined the top five contributing EEG channel dyads for the two significant components, *PC2* and *PC3*, as shown in Fig. 4. The respective loadings for each channel dyad for *PC2* and *PC3* are shown in Table 2. PC2 showed strongest loadings for Coherence and WPPC in fronto-parietal and centro-temporal networks. The top five contributing features included Fz–P8 Beta-band WPPC, Cz–T8 Theta-band WPPC, Cz–T8 Alpha-band WPPC, Fz–P8 Beta-band Coherence, and Cz–T8 Theta-band Coherence. This finding suggests a predominant role of centro-parietal and temporo-parietal synchrony linked to symptom dimensions captured by PC2. PC3 exhibited its strongest loadings for Coherence and WPPC in frontal, fronto-parietal, and fronto-temporal networks in multiple frequency bands. The five key contributing features were F7–P8 Theta-band Coherence, F7–P8 Theta-band WPPC, F7–T8 Beta-band WPPC, F7–Fz Gamma-band Coherence, and F7–P8 Delta-band Coherence. The prominence of F7 connections highlights the potential role of left frontal connectivity patterns in depressive symptom trajectories, particularly morbid ideation, for which PC3 showed significant associations.

### Post-hoc Analysis of Individual Dyads

To further elucidate the neurophysiological underpinnings of the significant principal components (PC2 and PC3) identified in the primary analyses, post-hoc models were conducted for their five most prominent EEG connectivity features. These dyads, reflecting high PCA loadings, were entered as predictors into LMEMs with CDRS total score and the CDRS morbidity subscore as the outcome variables. Separate models were fit for each dyad, including main effects of time (*Session*) and *Dyad* × *Session* interaction terms, and random intercepts for Subject. For PC2, none of the five top dyads *Cz–T8 Theta-band Coherence, Fz–P8 Beta-band WPPC*, *Cz–T8 Theta-band WPPC, Cz-T8 Alpha-band WPPC, Fz-P8 Beta-band Coherence* displayed significant main effect associations with *CDRS Total* scores or significant *Dyad* x *Session* interactions in their respective LMEMs, suggesting limited predictive value of the dyads in isolation. Similarly, there were no significant main effect associations with *CDRS Total* scores or significant *Dyad* x *Session* interactions for the top five loadings of PC3, comprising *F7–P8 Theta-band Coherence, F7–P8 Theta-band WPPC, F7-Fz Gamma-band Coherence, F7-P8 Delta-band Coherence, and F7-T8 Beta-band WPPC*. However, there was a trend level effect for *F7-T8 Beta-band WPPC (*β = −98.373, SE = 56.290, *t* = −1.748, *p* = 0.087).

## DISCUSSION

### General Findings

In this study, we investigated the longitudinal associations between depressive symptom severity, as indexed by the Children's Depression Rating Scale (CDRS), and EEG-derived functional connectivity measures in adolescents with major depressive disorder (MDD) using linear mixed effects models. We observed a significant decrease in CDRS total scores in the treatment course between baseline and week 4, with additional stabilization through week 16. This trajectory suggests that the majority of therapeutic improvement occurred early in treatment, involving antidepressant medication combined with evidence-based therapy (EBT). This is consistent with prior reports suggesting that the largest therapeutic response in pediatric depression typically occurs within the initial treatment window ^28^. These longitudinal changes provided the framework to test whether EEG-based functional connectivity measures track underlying neurophysiological mechanisms of treatment response in adolescent depression.

### Longitudinal Associations with Electrophysiological Changes – CDRS Total

Examination of associations between EEG signals and treatment-related changes in symptoms revealed significant time-dependent associations with CDRS total scores. Specifically, significant PC2 × Session and PC3 × Session interactions showed that higher baseline PC2 and PC3 values were linked to attenuated symptom improvement across the 16 weeks. In addition, the main effects of PC2 and PC3 on overall CDRS severity across sessions were only modest and reached trend-level significance, suggesting that these components moderate state-dependent longitudinal change rather than being static markers of symptom burden. Spatially, PC2 was characterized by positive loadings for coherence and WPPC in fronto-parietal and centro-temporal networks, particularly in beta and theta bands. PC3 was dominated by frontal and fronto-temporal loadings for Coherence and WPPC across multiple bands, with strong contributions from left frontal connections (F7-centered dyads). In adolescent MDD, neuroimaging studies have demonstrated that these same networks exhibit abnormal connectivity, with both hypoconnectivity and inefficient integration ^29^. These disruptions are associated with greater disease severity and executive dysfunction, implicating impaired top-down modulation and cognitive control as central electrophysiological features of depression in this population. These findings partially align with our previous findings of significantly lower theta band coherence in MDD patients versus healthy controls ^17^. Another study reported increased overall EEG coherence in fronto-temporal and parietal regions, particularly within the theta and beta frequency bands, characterizing a distinguishing feature of MDD across a broad age range ^30^. More specifically, our finding that functional connectivity within fronto-parietal and centro-temporal regions is associated with a weaker antidepressant response aligns with previous evidence showing that increased right fronto-temporal connectivity in the delta/theta frequency range differentiates adult non-responders from responders at week 8 of treatment ^31^. Also analogous to our present findings, an additional study demonstrated that dynamic functional connectivity between frontal and parietal regions predicted antidepressant response in adolescents, with higher connectivity associated with less symptom improvement ^32^. The involvement of fronto-parietal regions in MDD is further supported by fMRI studies showing that increased connectivity between the default mode network (DMN) and the left dorsolateral prefrontal cortex is associated with greater depressive symptom severity ^33,34^. Importantly, our study extends these prior findings by incorporating a comprehensive set of connectivity measures, Coherence, Imaginary Coherence, WPLI, and WPPC, within an adolescent MDD cohort, offering a more nuanced characterization of functional network dynamics during antidepressant treatment. In contrast, most previous investigations have focused primarily on coherence-based metrics and were conducted in adult populations ^9–12^.

### CDRS Subscale-Specific Findings

By extending analyses to CDRS subscores, we further refined the clinical relevance of EEG connectivity patterns. PC3 showed a robust interaction with Session for the CDRS Morbidity subscale, suggesting that this component not only tracked overall symptom severity but was specifically linked to the dimension of morbid thoughts. This suggests that PC3, which captured Coherence and WPPC in frontal, fronto-parietal, and fronto-temporal networks for multiple frequency bands, is a neurophysiological marker of a clinically salient subdomain of depression. Moreover, the prominence of F7 connections in these networks suggests the potential role of left frontal connectivity patterns as outlined above. These results suggest that while broad symptom improvement is reflected in CDRS total scores, subdomains such as morbidity may have unique neurophysiological correlates, with PC3 emerging as a candidate biomarker of this specific symptom dimension. The existing literature on EEG parameters predicting CDRS subscale scores is limited, with only a few studies reporting associations between DMN hyperactivity and excessive negative self-referential processing or thought rumination, which may involve morbid thinking ^35,36^. MDD is a highly heterogeneous condition, and global severity scores may obscure distinct symptom dimensions driven by different neural mechanisms. By examining subscales rather than overall MDD diagnostic scores, more specific patterns of dysfunction can be isolated, underlying circuitry disruptions and potentially contributing more directly to patients’ suffering.

### Role of Analyzed Frequency Bands

As shown in Fig. 4, the top loading dyad for both PC2 and PC3 includes the theta frequency band, while only one of PC3’s top five dyads features gamma coherence as a higher-frequency component. This pattern suggests that slower frequencies, particularly in the theta, delta, and alpha bands, seem to primarily drive the observed longitudinal associations between functional connectivity measures and depressive symptom change. Biologically, coherence within lower frequency bands has been proposed to reflect long-range cortical integration and subcortical-cortical communication, indexing large-scale neural network synchrony and default mode network regulation relevant to the pathophysiology of depression ^33^. In line with this, our group previously reported reduced theta-band coherence and increased delta power in adolescents with MDD compared with healthy controls ^17^. Similarly, another study found higher delta, alpha, and beta coherence in patients with suicidal behavior compared to patients with suicidal ideation ^37^, underscoring the clinical relevance of low-frequency synchronization. Together, these findings suggest that functional connectivity measures, particularly in lower frequency bands, may hold diagnostic value for distinguishing individuals with depression from healthy controls and may also be associated with the severity of depressive symptoms.

### Limitations and Future Directions

There were several limitations associated with our exploratory study. First, the sample size of our study was relatively small (n = 21), which may limit the generalizability of our findings. Replication in larger independent cohorts is necessary to validate these results externally and ensure their robustness across developmental stages and clinical subgroups. Second, this study was naturalistic in design and did not include a control or placebo group. We therefore cannot determine whether the observed EEG connectivity changes are entirely specific to antidepressant response or may represent non-specific clinical improvements or time-related effects, necessitating future studies with placebo arms or active comparators. Also, participants received varying combinations of evidence-based treatments, including fluoxetine or escitalopram alongside psychotherapy. As such, we were unable to isolate the specific contributions of medication versus psychotherapy, compare antidepressant classes, or evaluate differential treatment effects. Moreover, detailed standardization of treatment parameters, including dose trajectories and adjustments, adherence, concomitant medications, and adverse effects, was beyond the scope of our study. Third, while PCs effectively capture latent patterns of variance in EEG connectivity, reducing 1440 EEG connectivity features into orthogonal components, their physiological interpretation remains indirect. Although PCA captures shared variance, it does not establish mechanistic causality. Future work combining multimodal neuroimaging, such as fMRI and MEG, could clarify the extent to which the identified EEG-derived components reflect dysregulation within fronto-parietal and limbic control networks implicated in adolescent depression. Importantly, our model was not designed to predict treatment response or compare against placebo, but rather to track longitudinal changes in functional connectivity and their association with symptom severity over time in adolescents. Longitudinal trajectories of qEEG dynamics in adolescent MDD still remain understudied, and our present findings provide preliminary evidence that distributed EEG connectivity patterns track symptom trajectories in real-world settings. Future research should incorporate larger, prospectively controlled cohorts with more detailed characterization of dosages and concomitant treatments. Prior adult studies have shown the promise of EEG-based predictive modeling. For instance, Wu et al. developed the SELSER computational model that could predict symptom improvement as a result of treatment with sertraline, indicating a specific neurobiological signature for sertraline response, showing cross-site generalizability ^12^. Similarly, a meta-analysis by Watts et al. (2022) reported high pooled accuracy of 83.9% for EEG-based machine learning models predicting treatment response in MDD ^11^. Such predictive frameworks illustrate a pathway toward personalized psychiatry and emphasize the importance of reproducible biomarkers in adolescent depression.

## CONCLUSION

This study highlights the potential of EEG-based functional connectivity measures as putative biomarkers for treatment response in adolescent MDD. Longitudinal changes in specific EEG networks correlated with symptom improvement, underscoring the importance of non-invasive neurophysiological measures in psychiatry. Taken together, our findings suggest that EEG-derived biomarkers of treatment response in adolescent depression are likely to be spatially distributed, frequency-specific, and nonlinear in nature. The combination of PCA-based dimensionality reduction and longitudinal LMEMs offers a promising analytic pipeline to isolate functionally and clinically relevant brain network features. Our findings suggest that EEG connectivity–derived PCs hold promise as state-sensitive markers of treatment response in adolescent depression. In our analysis, PC2 primarily reflected distributed fronto-parietal synchrony, while PC3 captured frontal and fronto-temporal coherence patterns, including those linked to morbid ideation. The multivariate and distributed nature of clinically relevant neural signatures underscores the advantage of dimensionality reduction approaches like PCA, which capture latent patterns not evident in isolated channel pairs. Future studies using larger, prospectively controlled cohorts with standardized treatment parameters should integrate multimodal neuroimaging, such as fMRI and MEG, to validate whether the identified PCs reflect dysfunction in fronto-parietal and fronto-temporal networks and to disentangle medication, psychotherapy, and time-related effects.

## Figures and Tables

**Table 1:** Demographics of overall sample

Age, mean (SD)	MDD Cohort (n = 21)
	15.71 (1.15)
Sex, N (% female)	18 (86)
Race	
White	19
African American	1
Other	1
CDRS-R total score, mean (SD)	63.52 (12.42)
CDRS Morbidity, mean (SD)	2.57 (1.80)
CDRS Suicidality, mean (SD)	2.81 (1.57)
CDRS Difficulty having fun, mean (SD)	4.57 (1.12)
CDRS Social withdrawal, mean (SD)	4.10 (1.48)

**Table 2:** Principal component loadings

Feature	Loading Value
PC2	
Cz_T8_Theta_Coherence	−0.0666
Fz_P8_Beta_WPPC	−0.0658
Cz_T8_Theta_WPPC	−0.0652
Cz_T8_Alpha_WPPC	−0.0651
Fz_P8_Beta_Coherence	−0.0645
PC3	
F7_P8_Theta_Coherence	−0.0687
F7_P8_Theta_WPPC	−0.0677
F7_Fz_Gamma_Coherence	−0.0607
F7_P8_Delta_Coherence	−0.0606
F7_T8_Beta_WPPC	−0.0596
